# Parent Report of Community Psychiatric Comorbid Diagnoses in Autism Spectrum Disorders

**DOI:** 10.1155/2011/405849

**Published:** 2011-08-18

**Authors:** Rebecca E. Rosenberg, Walter E. Kaufmann, J. Kiely Law, Paul A. Law

**Affiliations:** ^1^Department of Medical Informatics, Kennedy Krieger Institute, Painter Building, First Floor, 3825 Greenspring Avenue, Baltimore, MD 21211, USA; ^2^Center of Genetic Disorders of Cognition and Behavior, Kennedy Krieger Institute, 716 N. Broadway, Baltimore, MD 21205, USA; ^3^The Johns Hopkins University School of Medicine, Baltimore, MD 21205, USA

## Abstract

We used a national online registry to examine variation in cumulative prevalence of community diagnosis of psychiatric comorbidity in 4343 children with autism spectrum disorders (ASD). Adjusted multivariate logistic regression models compared influence of individual, family, and geographic factors on cumulative prevalence of parent-reported anxiety disorder, depression, bipolar disorder, and attention deficit/hyperactivity disorder or attention deficit disorder. Adjusted odds of community-assigned lifetime psychiatric comorbidity were significantly higher with each additional year of life, with increasing autism severity, and with Asperger syndrome and pervasive developmental disorder—not otherwise specified compared with autistic disorder. Overall, in this largest study of parent-reported community diagnoses of psychiatric comorbidity, gender, autistic regression, autism severity, and type of ASD all emerged as significant factors correlating with cumulative prevalence. These findings could suggest both underlying trends in actual comorbidity as well as variation in community interpretation and application of comorbid diagnoses in ASD.

## 1. Introduction

Diagnoses of autism spectrum disorders (ASD) are increasing [1, 2]. This trend is accompanied by an evolving discussion about codiagnosis and treatment of associated psychiatric disorders. Such disorders may affect up to 70% of children with ASD at any given time [3, 4], and up to 100% over a lifetime in many small clinical studies (*n* ≤ 200) [4–8]. This compares with 35% by adolescence in the general population [9] and more so than their non-ASD peers with isolated ID [10, 11]. The data in these studies, applying standardized research diagnoses, are helpful for elucidating etiology, phenotypes, and eventually targeted therapies. 

However, little is known about actual patterns of community-assigned comorbid diagnoses in ASD. Understanding how various factors are associated with community-diagnosed psychiatric comorbidity in ASD has important implications for identification of overall trends in community practice, variation in health services, and management of expectations among clinicians and families. 

First, comparison of overall cumulative prevalence in the community compared with research-assigned diagnoses in cumulative prevalence studies will help identify variation in application and community interpretation of current DSM IV-TR [12] guidelines. Previous clinic-based investigations have used more precise research-assigned comorbid diagnoses in ASD (albeit with tools that vary between studies [3, 13–16]). These have been primarily for point prevalence estimates of variation [3, 6, 17–22] of specific comorbidity within the ASD and by ID status and gender [3, 23, 24]. By examining actual diagnoses among a large national US sample of a wide variety of children with ASD, we may be able to identify trends in diagnosis receipt and promote standardization tools to reduce discrepancies and variation in ASD care. 

Second, variation in community diagnosis by environmental factors, such as geographic features, is a reflection of health care quality and delivery patterns in reallife settings. Previous studies have reported considerable variation in timing and receipt of ASD diagnosis in the community. This also includes psychotropic medication use in ASD, associated factors including presence of ID, sex, ethnicity, race, family socioeconomic status (SES), US region, urbanicity, and secular trends [25–28]. Analysis of these differences in cumulative prevalence of community-diagnosed psychiatric comorbidities in ASD will help identify deficits in recognition of ASD comorbidities among various subgroups, such as mood disorders in children with AS [24]. Community diagnostic trends reflect real-world situations and thus are useful for public policy considerations.

Finally, but perhaps most importantly, community providers and families could benefit from more information predicting which children are more likely to receive comorbid psychiatric diagnoses, and thus they could anticipate these conditions. Multivariate analysis of lifetime likelihood of comorbid diagnosis is possible with an existing nationwide database, albeit by parent report rather than clinical diagnosis. Various profiles may emerge from such analyses to prompt clinicians and families to recognize symptoms as part of a potentially treatable and perhaps undiagnosed comorbidity rather than attributing features to the ASD alone. This diagnostic bias, first described in the 1980s in the intellectually disabled population, is called “diagnostic overshadowing” [29, 30]. Little information exists about diagnostic overshadowing in the ASD population, and this study will therefore provide some information about variation in the field and suggest further research and variables that influence this important bias.

Until recently, no large-scale surveys of lifetime community diagnoses have been available. Since 2006, the creation of the largest online voluntary autism registry of parent reported data, the Interactive Autism Network (IAN), has provided valuable information on community trends in ASD among over 14,000 affected children and more than 22,000 family members. We therefore used parent report of lifetime diagnoses to assess variation of community-diagnosed psychiatric comorbidity in ASD. In particular, we wished to examine potential factors associated with community diagnosis of psychiatric comorbidity, including gender, type of ASD, ASD severity, history of autistic regression, and ID status. This was done while controlling for external factors like ethnicity, race, maternal education, and history of psychiatric illness, and geographic variation.

## 2. Method

### 2.1. Participants

IAN is an online US-based research database begun in April 2007, with more than 35,000 individuals enrolled. It includes more than 12,000 children younger than 18 with an ASD and their immediate family members. All data are voluntarily submitted by families. The database is continually updated and recruitment is ongoing. A pilot phase of data collection began on September 11, 2006. 

The current analysis was conducted with data extracted on February 20, 2009 from the database of all participants aged 5 to 18 years who had completed primary history questionnaire by that time (4848). We excluded those with an initial diagnosis of childhood disintegrative disorder (*n* = 2) or with a non-DSM-IV-TR diagnosis (generic “autism spectrum disorder” or generic “pervasive developmental disorder”); (*n* = 503). The final study sample therefore included 4343 participants.

### 2.2. Measures

#### 2.2.1. IAN Questionnaires

The IAN project data collection consists of multiple topic-specific forms, authored by the IAN research team in collaboration with other researchers. Questionnaires are available at http://www.iancommunity.org/cs/ian_research_questions/ian_research_questions/. All families complete the initial registration and then are invited to complete several other questionnaires including a profile on each affected child. Once registered, families receive reminders every 2 weeks to complete outstanding questionnaires. These surveys were developed by IAN staff in collaboration with members of the IAN Science Advisory Committee, were piloted with families, and were revised as needed. All participants completed a second-generation version of the questionnaires. 

Our dependent variables were parent-reported history of any anxiety disorder, depression, bipolar disorder (manic/depressive disorder), attention deficit/hyperactivity disorder (AD/HD), or attention deficit disorder (ADD), or schizophrenia. Although diagnosis of AD/HD and ADD are not “permitted” by current DSM IV-TR criteria for ASD, we included these because many children with ASD display signs of AD/HD and ADD and often receive these diagnoses in the community setting. For each disorder, families were asked, “Has [your child] ever been diagnosed with or treated for [disorder]?” For anxiety disorder, the following information was provided below the question: “Anxiety disorders include panic disorder/panic attacks, obsessive compulsive disorder, posttraumatic stress disorder, social anxiety disorder (social phobia), specific phobias, and generalized anxiety disorder.”

Independent variables extracted from the IAN database questionnaire responses included current ASD diagnosis, race, gender, birth date, initial evaluator, history of regression, and current address. The race variable corresponded to categories delineated by the US Census Bureau [31]. For the variable of ID status, participants were broadly categorized as ID if they *either *(a) reported ever receiving a diagnosis of “mental retardation” or (b) reported an IQ score of <70, if available. “History of regression” was defined as reported moderate to severe communication or social skill loss before the age of 3. Initial evaluator was condensed from ten possible choices into two categories, “Psychiatrist/psychologist” and “Other,” based on exploratory data analysis. 

Regions were defined as Midwest: Indiana, Iowa, Illinois, Kansas, Michigan, Minnesota, Montana, North Dakota, Nebraska, Ohio, South Dakota, Wisconsin; Northeast: Connecticut, Delaware, Massachusetts, Maryland, Maine, New Hampshire, New York, New Jersey, Pennsylvania, Rhode Island, Vermont; South: Alabama, Arkansas, DC, Florida, Georgia, Kentucky, Louisiana, Mississippi, Oklahoma, Tennessee, Texas, North Carolina, South Carolina, Virginia, West Virginia; West: Alaska, Arizona, California, Colorado, Hawaii, Idaho, Montana, New Mexico, Nevada, Oregon, Utah, Washington, Wyoming.

Data on maternal education and psychiatric history were extracted from IAN surveys for 4055 out of 4343 participants (93.7%). Only 10% of biological fathers completed forms; therefore, no paternal data were included. Maternal education history was analyzed by four categories: no high school completion; high school diploma, no college; some college; college diploma. History of maternal psychiatric illness was positive if mothers reported “yes” to current/past history of diagnosis or treatment for any anxiety disorder, depression, bipolar disorder, AD/HD or ADD, or schizophrenia.

#### 2.2.2. Social Responsiveness Scale

The Social Responsiveness Scale (SRS) [32] is a validated 65-item, parent/teacher-completed, norm-referenced tool designed to discriminate between individuals with and without ASD. The SRS emphasizes “social deficits,” in particular deficits in social reciprocity, based on the Autism Diagnostic Interview-Revised [33]. Clinical *T *score screening cutoffs of <55, 55–59, 60–75, and >75 suggest likely unaffected status and borderline, mild-moderate, or severe autistic features, respectively. The SRS parent form was offered to all IAN participants with children ≥4 years and <18 years as of February 2008. Among the final sample of 4343, 2373 (54.6%) completed the SRS.

All survey data were entered by parents and maintained in the Internet Mediated Research System, IMRS (MDLogix, Baltimore, MD). Electronic consent was elicited from participating families under the auspices of the Johns Hopkins Medicine Institutional Review Board (#NA_00002750). If families skipped a question based on an answer to the previous question, answered “Do not Know,” or declined to answer a question, data were recorded as missing. Within individual analyses, we used the modelwise approach to handling missing data. Those individuals without complete data were dropped from the specific analysis rather than imputed or estimated.

### 2.3. Data Analysis

Analyses were performed using STATA 9.2 (College Station, TX). For the descriptive analyses, chi-square analysis and ANOVA were used for categorical and continuous independent variables, respectively. Due to small cell size, two-tailed Fisher's exact test was used in bivariate race and maternal education analyses. To assess correlation of comorbidities to each other, we used logistic regression for each comorbidity, adjusted by age. We then used multivariate logistic regression to examine the independent effects of the following *a priori* risk factors on history of each psychiatric comorbidity: gender, age, current ASD diagnosis, SRS score, ID status, history of regression, race, ethnicity, maternal education and psychiatric history, type of initial evaluator, and region.

## 3. Results

Table 1 shows all patients in the cohort.

Participants were more likely to have a single psychiatric comorbidity (26.9%) than two (14.4%), three (6.3%), or four (1.5%) comorbidities (data not shown). Of those with at least one comorbidity, 45.2% had two or more. 

The most common comorbid diagnosis was AD/HD or ADD (38.1%), followed by anxiety disorders (26.2%), depression (11.0%), and bipolar disorder (5.2%), as seen in Table 2. Overall, only 23/4343 (0.5%) reported a diagnosis of schizophrenia, and thus this disorder was not included in the analysis. 

Table 2 shows significant independent variables (*P* < .01) on bivariate analysis by child/family characteristic for each reported psychiatric comorbidity.

There was no difference in proportion of overall comorbidity by gender on bivariate analysis. However, older age, diagnosis of AS, higher SRS score (when available), presence of ID, lack of autistic regression, non-Hispanic ethnicity, initial evaluation by a psychiatrist or psychologist, and history of maternal psychiatric illness were all associated with increased risk of psychiatric comorbidity (chi-square *P* < .01). 

Bivariate chi-square analysis of comorbidity by race was significant only for anxiety (*P* < .02). Hispanic children were less likely to have an anxiety or depression diagnosis, compared with their non-Hispanic peers (*P* < .01). Chi-square analysis of depression showed that children of women with college education but not graduate degrees were more likely than other children to have a depression diagnosis (13.0% versus 9.5% and 10.0%, *P* = .03). Children of college-educated mothers were less likely to have bipolar disorder diagnosis (*P* = .011).

As shown in Table 3, logistic regression of anxiety, depression, and bipolar disorder cooccurrence were high among those with at least one comorbidity, adjusted for age, *P* < .001); the odds of having any anxiety disorder was two times higher for those with depression compared to those without depression, for example. AD/HD or ADD, however, was negatively correlated with anxiety (OR 0.1, 95% CI, 0.1, 0.2) and depression (OR 0.7, 95% CI, 0.5, 0.9).

Table 4 shows the results of five separate multivariate logistic regressions for any comorbidity and separately for anxiety, depression, bipolar disorder, and AD/HD or ADD, adjusting for maternal education, ethnicity, initial evaluator, location, and maternal psychiatric illness. 

Female gender was moderately protective for both bipolar disorder (OR 0.5, 95% CI, 0.3, 0.9) and for AD/HD or ADD (OR 0.8, 95% CI, 0.6, 1.0). PDD-NOS and AS were significantly correlated with increased odds of each comorbidity and for overall comorbidity. SRS score was positively correlated with increased odds of each and overall comorbidity (*P* < .001). For every 10-point increase in SRS score, for example, odds of any comorbidity was at least 20% higher (OR 1.2, 95% CI, 1.1, 1.3) than for those without comorbidity. Presence of ID was not associated with any comorbidity except as a negative correlate of depression (OR 0.6, 95% CI, 0.4, 0.9; *P* < .01). Autistic regression was associated with lower risk of any comorbidity (OR 0.8, 95% CI, 0.6, 1.0), any mood disorder (OR 0.7, 95% CI, 0.5, 1.0), and AD/HD or ADD (OR 0.7, 95% CI, 0.5, 0.8).

## 4. Discussion

To our knowledge, this is the largest study of factors associated with community-assigned lifetime diagnosis of psychiatric comorbidities among children with ASD (*N* = 4343). In contrast to prior practice-based and epidemiologic studies, we were able to use multivariate analysis to find evidence of both individual and environmental factors influencing overall risk of community diagnosis of psychiatric comorbidities. In particular, this information about factors associated with comorbid diagnosis at the community level is important in assessing evaluator trends as well as specialty access issues for children with ASD. Overall, we did confirm previous research suggesting that psychiatric comorbidity, whether point- or cumulative prevalence, is increased in children and adolescents with ASD compared with general population estimates, including risk of multiple comorbidities [3, 22, 34]. Lifetime prevalence of psychiatric disorder in the general population, by age 16, was approximately 37% [9] while among our population of 5–18 y, this figure was 49%.

### 4.1. Autistic Severity, Type of ASD, and Intellectual Disability

Earlier research suggested that type and severity of ASD influence risk of psychiatric comorbidity [17, 35]. Our study found that diagnoses of PDD-NOS and AS were associated with increased cumulative prevalence, relative to AD, of any and every type of psychiatric comorbidity. This was true even when adjusting for other variables including social impairment using SRS. This is consistent with findings of a state-wide cohort study of children with ASD (*n* = 586) using psychiatric comorbid diagnoses from community mental health centers [36]. That study found significantly higher proportions of AD/HD and mood disorder diagnoses in those with PDD-NOS or AS compared with AD. However, this sample was likely biased toward more complicated cases and represented only a small subsection of children with ASD in that state. 

Our community-based findings differed from those reported by Simonoff et al., who did not find a univariate association between type of clinician-confirmed ASD diagnosis and point prevalence of psychiatric disorder in their epidemiologic sample of 112 participants [3]. Similarly, Kim et al. did not find significant univariate differences by cumulative comorbidity prevalence by ASD subtype among high-functioning subjects in a practice-based follow-up cohort [7]. In addition, while we found that increased autistic severity was positively correlated with cumulative psychiatric comorbidity diagnosis in the community, research-based point prevalence estimates have not [3].

These differences between research- and clinician-confirmed diagnosis and our community-based findings suggest that recognition of psychiatric comorbidity is highly variable. Perhaps those with AS and PDD-NOS, with higher average IQ and lower autistic severity, present with more “typical” signs and symptoms of affective disorders which are more readily recognized by evaluators and subsequently receive a diagnosis. Clinicians need to be aware that psychiatric comorbidity should be carefully considered in all patients with ASD [4]. Further research about the effects of diagnostic overshadowing in the ASD population, as has been documented for the ID population [29, 30], is clearly warranted. However, without standardized scales for assessment of psychiatric comorbidity in ASD, this level of care is difficult to find in the nonresearch setting. 

Somewhat reassuringly, though, our study suggests an equally important *lack* of variation with ID and psychiatric comorbidity in ASD. This is consistent with the point-prevalence findings of both Simonoff et al. and Brereton et al. that overall, those with both ASD and ID were just as likely to report psychiatric comorbidity as those without ID [3, 10]. A small subanalysis of an epidemiologic ID sample, using a screen for PDD, suggested no difference in psychiatric comorbidity for those with “positive” PDD screens versus negative, except for increased anxiety scores, largely due to repetitive thoughts and behaviors [37]. From these parent-reported data, ID is not “protective” of most psychiatric comorbidity, with the exception of depression, as previously reported [4, 38]. However, this could reflect lack of recognition rather than true lack of prevalence. The ID data do not reflect actual adaptive functioning, and parents may not accurately report IQ. Therefore, more careful examination at the intersection of ID, ASD, and psychiatric comorbidity is certainly needed.

### 4.2. Autistic Regression

To our knowledge, autistic regression has not previously been examined as a factor affecting psychiatric comorbidity in ASD in any type of analysis. Regressive autism may be a particular subtype of ASD [39], related to decreased functional and verbal ability [40]. Decreased risk for any comorbidity for those with reported history of regression, compared with nonregressors, requires further corroboration, especially given the potential confounding with intellectual disability.

### 4.3. Demographic Influences

In the general population, affective disorders are more common in girls than boys by about 30%, and AD/HD or ADD (especially AD/HD) is approximately five-times more often diagnosed in boys [9]. There was a surprising lack of correlation with female gender and community psychiatric comorbid diagnosis in our national voluntary sample, although others have suggested a link previously [41]. We did not find either of these differences in this ASD sample, concurring with two other large studies of clinical diagnoses [3, 10]. In fact, in our study, female gender was protective for bipolar disorder, and girls were only 25% less likely than boys to have AD/HD or ADD. Because we controlled for specific ASD diagnosis, age, and ID, this *lack* of difference by gender suggests that presence of ASD overcomes gender differences in community-assigned psychiatric comorbid diagnosis. 

Race was not associated with lifetime community diagnosis of psychiatric comorbidity in multivariate analysis. However, Hispanic ethnicity was a significant negative correlate in bivariate analysis, possibly due to cultural differences in perception of acceptable behavior [11] or evaluator bias or other health system issues. However, the lack of finding in multivariate analysis could suggest that other factors are more important. 

Cumulative prevalence of psychiatric comorbidity, not surprisingly, increased with age. The effect of secular trends—that is, changes in clinical practice trends over time—on community psychiatric diagnosis may indeed have a role distinct from the effects of increased exposure (increasing age) to developing a condition. We cannot, however, tease apart these differences without knowing the age of receipt of psychiatric comorbid diagnosis, as in this dataset. Future prospective studies of the ASD population are clearly needed to understand the natural history of these disorders and help families and clinicians anticipate such developments.

### 4.4. Geographic and Environmental Influences

Finally, based on our past research of external factors influencing diagnostic trends [25], it was not surprising that evaluator, location, maternal characteristics, and ethnicity are important variables in community diagnosis of psychiatric comorbidity. Initial ASD diagnosis by a psychiatrist or psychologist was correlated with psychiatric comorbid diagnosis [42]. However, we lack data about what type of evaluator actually gave the comorbid diagnosis or when it was given. More detailed data may be able to discern if it is merely the more complicated cases who present to psychologists and psychiatrists initially (i.e., the Berkson effect) [42]. Conversely, this discrepancy may be explained by differential access (by geography or socioeconomic status) to specialty pediatric mental health professionals who are more likely to screen for comorbidities. Future IAN questionnaires will solicit more specific information from families, but maternal education (our proxy for socioeconomic status) did not persist as a factor with psychiatric comorbidity in multivariate analysis. 

Our data on geographic differences in community-reported diagnoses confirm that the current diagnostic criteria of psychiatric comorbidity are variably interpreted or inconsistently applied [3, 13–16]. The evolution of diagnostic criteria in both ASD and psychiatric comorbidity in children and adolescents, as anticipated in the DSM V, will hopefully help primary care clinicians recognize, diagnose, and treat psychiatric comorbidity in ASD. In particular, clinicians are currently limited in assigning a diagnosis for the AD/HD and ADD symptoms that often accompany ASD, since current DSM IV-TR guidelines “prohibit” assignment of AD/HD or ADD as a concurrent diagnosis in ASD. With the high prevalence of these comorbid diagnoses now (i.e., 38%), future DSM guidelines should reflect this reality. As well, given limited access to pediatric mental health specialists in many parts of the US, the onus for ASD management will increasingly fall to primary care providers who would benefit from clarification and accessible tools and guidelines in a currently unstandardized climate [4].

The important and complex role of family psychiatric history also is critical to understanding both comorbidity and overall ASD heritability patterns. It may also be related to seeking of and recognition of comorbid diagnoses by affected parents. We are exploring the nature of this association in a separate analysis.

### 4.5. Limitations

This study is the largest of factors associated with community diagnoses of psychiatric comorbidity in ASD but has some limitations. Professional diagnosis of ASD in the IAN database has been clinically validated [43] and parent-report verified [44]. Thus there is reason to believe that parent report on professional diagnosis of comorbidities is also reasonably reliable. Therefore, we cannot validate presence or absence of psychiatric comorbidity against a research standard or estimate true prevalence. However, we are able to compare parent-reported diagnosis among IAN participants (by type of ASD, gender, etc.) and to examine actual experience of ASD in the community. Future research is urgently needed to consistently define psychiatric comorbidity within ASD and promote subsequent assessment. Availability of comorbidity screening and diagnostic tools as Internet forms may further validate these community-assigned diagnoses.

IAN is a web-based registry of parent-reported information and, as a newer form of data collection, is gaining acceptance as a reliable and valid form of Internet-collected data. Current research supports Web-based surveys of medical information as a reliable means of data collection [45]. This has been demonstrated by a similar registry for a related disorder, Rett syndrome [46], and in other studies using IAN data [47]. 

Our ID data, in particular, may be biased toward underestimation due to parent report. We designated positive ID status to those who reported an IQ < 70, regardless of self-reported ID. However, we do not know which IQ measure was used and at what age. 

There is a small bias within IAN toward more educated and less ethnically diverse families [48], but we have assumed that these biases are constant throughout this sample of convenience. Nevertheless, the sample may be somewhat unrepresentative of this clinical population. 

Other specific issues in data interpretation are unique to the data collection method for lifetime rather than current diagnosis in IAN. Parents soon will be able to report on current and past diagnoses with potential for updating. Because IAN continuously collects data, we hope to maximize the potential for longitudinal studies of community-assigned psychiatric comorbid diagnoses, including collecting more specific comorbidity data.

Finally, there is the problem of “multiplicity,” in that numerous statistical comparisons were conducted here, with alpha set at  .01. Therefore, some of the significant associations identified were almost certainly spurious.

## 5. Conclusion

Despite the limitations discussed in the previous section, there are important implications related to these findings as they reflect trends in application of comorbid psychiatric diagnosis in ASD across US communities. The discrepancy between research- and community-assigned variation in psychiatric comorbidity by type and severity of ASD suggests a large and important gap and likely underdiagnosis of potentially treatable conditions. Further investigation of differences in “diagnosis risk” by autistic regression status and lack of differentiation by gender is also warranted. The comprehensive diagnosis and treatment of more children with ASD in community and educational systems requires urgent standardization and guidelines for optimal outcomes [1].

##  Disclosure

This paper was supported by Autism Speaks. The funder did not have a direct role in this paper. The authors have no other disclosures to report.

## Figures and Tables

**Table 1:** Characteristics* of children with ASD, aged 5–18 y by parent-reported lifetime history of psychiatric comorbidity (*n* = 4343).

	No psychiatric comorbidity	Any psychiatric comorbidity^†^	*P* value (chi-square or Wilcoxon rank)
*N* (%)	2213 (50.9)	2130 (49.1)	
Gender, %			
Male (*n* = 3625)	50.9	49.1	.861
Female (*n* = 721)	51.3	48.9	
Median age (y)	8.9	11.7	<.001
Current autism spectrum disorder**, %			<.001
Autism/autistic disorder (*n* = 2161)	62.9	37.1	
PDD-NOS (*n* = 1024)	50.2	49.9	
Asperger syndrome (*n* = 1158)	29.3	70.8	
Adjusted SRS Score (*n* = 2373)	85.2	88.0	<.001
Intellectual disability**, %			<.001
Not reported (*n* = 3156)	52.1	47.9	
Present (*n* = 1170)	47.4	52.6	
History of specific skill loss^a∗∗^, %			<.001
No loss (*n* = 2930)	47.9	52.9	
Skill loss (*n* = 1413)	59.3	41.1	
Race			.663
White (*n* = 3967)	50.7	49.3	
Black/African-American (*n* = 139)	54.0	46.0	
Asian American (*n* = 54)	55.6	44.4	
Other (*n* = 183)	53.6	46.5	
Ethnicity**, %			.004
Hispanic (*n* = 330)	58.5	41.5	
Not Hispanic (*n* = 4013)	50.3	49.7	
Maternal education level, %			.720
High school or less (*n* = 502)	51.8	48.2	
Some college (*n* = 1561)	49.7	50.3	
College graduate (*n* = 1987)	50.2	50.0	
Maternal psychiatric history, % (*n* = 4055)			<.001
No reported history (*n* = 1802)	59.6	40.4	
History of psychiatric illness^b^ (*n* = 2253)	42.7	57.3	
Initial evaluator** %			<.001
Psychiatrist or psychologist (*n* = 1343)	39.2	60.8	
Other (*n* = 2999)	56.2	43.8	
Region**, %			<.001
West (*n* = 725)	55.9	44.2	
All other regions (*n* = 3618)	50.0	50.0	

AD/HD or ADD = attention-deficit/hyperactivity disorder or attention-deficit disorder, respectively; SRS = Social Responsiveness Scale.

^a^Moderate-severe loss of communication and/or social skills between ages 12 and 36 months.

^b^Includes any anxiety disorder, depression, bipolar disorder, AD/HD or ADD, and/or schizophrenia.

^†^Any of the following comorbidities reported at the time of data collection: anxiety disorder, depression, bipolar disorder, AD/HD or ADD, or schizophrenia.

--23/4343 had history of schizophrenia (0.5%).

**Table 2:** Characteristics of children with ASD by presence of ≥1 psychiatric comorbidity, aged 5–18 y, significantly different from peers without particular comorbidity (*P* < .01 by chi-square).

	Proportion by reported history of psychiatric diagnoses			
Child characteristic	Any anxiety disorder (*n*)	Depression (*n*)	Bipolar disorder (*n*)	AD/HD or ADD (*n*)
Proportion reported in total sample (*n* = 4343)*	26.2 (1136)	11.0 (477)	5.2 (222)	38.1 (1653)

Current age category (y), *n *				
5–8 (*n* = 2276)	15.6 (356)	2.5 (57)	2.3 (52)	26.9 (611)
9–12 (*n* = 1271)	33.4 (424)	15.7 (200)	6.9 (87)	48.0 (610)
13–18 (*n* = 796)	44.7 (356)	27.6 (220)	10.4 (83)	54.3 (432)

Current autism spectrum disorder, %				
Autism/autistic disorder (*n* = 2161)	19.0 (410)	4.8 (104)	3.0 (65)	26.5 (572)
PDD-NOS (*n* = 1024)	25.2 (258)	8.3 (85)	5.6 (57)	39.8 (407)
Asperger syndrome (*n* = 1150)	40.4 (468)	24.9 (288)	8.6 (100)	58.2 (674)

SRS *t* score category, % (*n* = 1171)				
<55 unaffected (*n* = 46)	21.7 (10)	6.5 (3)	4.4 (2)	23.9 (11)
55–59 borderline (*n* = 47)	12.8 (6)	6.4 (3)	2.1 (1)	34.0 (16)
60–75 mild/moderate (*n* = 432)	18.8 (81)	6.3 (27)	1.6 (7)	37.0 (160)
>75 severe (*n* = 1843)	27.7 (511)	12.0 (254)	5.5 (101)	39.3 (724)

Intellectual disability, %				
Not reported (*n* = 3156)	25.1 (793)	12.0 (380)	4.7 (147)	37.4 (1181)
Present (*n* = 1170)	29.2 (342)	8.2 (96)	6.3 (74)	40.1 (469)

History of specific skill loss ^a^, %				
No loss (*n* = 2930)	28.0 (820)	13.1 (384)	5.9 (173)	42.4 (1241)
Skill loss (*n* = 1413)	22.4 (316)	6.6 (93)	3.5 (49)	29.2 (412)

Ethnicity, %				
Hispanic (*n* = 330)	19.1 (63)	11.3 (455)	3.3 (11)	32.1 (106)
Not Hispanic (*n* = 4013)	26.7 (1073)	6.7 (22)	5.3 (211)	38.6 (1547)

Maternal psychiatric history, % (*n* = 2017)				
No reported history (*n* = 1798)	20.5 (369)	6.4 (115)	2.9 (52)	30.5 (549)
History of psychiatric illness^b^ (*n* = 2253)	31.7 (713)	15.3 (345)	6.9 (156)	44.7 (1008)

Initial evaluator %				
Psychiatrist or psychologist (*n* = 1343)	33.8 (454)	18.0 (242)	7.8 (105)	49.0 (658)
Other (*n* = 2999)	22.7 (682)	7.8 (235)	3.9 (117)	33.1 (994)

*Note: Individuals with multiple comorbidities are counted separately for each comorbidity.

AD/HD or ADD = attention-deficit/hyperactivity disorder or attention-deficit disorder, respectively; SRS = Social Responsiveness Scale.

Comorbidity did not significantly vary (*P* > .01) by gender, region, or maternal education.

^a^Moderate-severe loss of communication and/or social skills between ages 12 and 36 months.

^b^Includes any anxiety disorder, depression, bipolar disorder, AD/HD or ADD, and/or schizophrenia.

^†^Any of the following comorbidities reported at the time of data collection: anxiety disorder, depression, bipolar disorder, AD/HD or ADD, or schizophrenia.

--23/4343 had history of schizophrenia (0.5%).

**Table 3:** Odds ratio (OR) of lifetime history of comorbidity among study participants with ASD, with at least one psychiatric comorbidity (*n* = 2133).

Diagnosis	OR (95% CI) any anxiety disorder	OR (95% CI) depression	OR (95% CI) bipolar disorder
Depression	2.0 (1.6, 2.5)**	—	—
Bipolar disorder	1.7 (1.2, 2.2)**	3.3 (2.4, 4.4)**	—
AD/HD or ADD	.1 (.1,.2)**	.7 (.5, .9)**	1.0 (.7, 1.5)*

**P < .001  *P = ns (.792).

AD/HD or ADD = attention-deficit/hyperactivity disorder or attention-deficit disorder, respectively.

**Table 4:** Multivariate logistic regression odds ratios (95% CI) of overall and individual parent-reported lifetime psychiatric comorbidities in ASD (*n* = 2219), by statistically significant factor (*P* < .001 unless otherwise noted).

	Any comorbidity	Any anxiety disorder	Any mood disorder (depression and/or bipolar disorder)	Depression	Bipolar disorder	AD/HD or ADD
Female Gender	ns	ns	ns	ns	.5 (.3, .9)*	.8 (.6, 1.0)*

Autism spectrum diagnosis (reference: autistic disorder)						

PDD-NOS	2.1 (1.6, 2.6)	1.9 (1.5, 2.5)	2.8 (1.9, 4.2)	2.3 (1.5, 3.7)	3.6 (2.0, 6.3)	2.1 (1.6, 2.6)
Asperger	2.8 (2.2, 3.5)	2.5 (1.9, 3.3)	3.9 (2.7, 5.7)	3.5 (2.3, 5.1)	2.9 (1.6, 5.1)	2.4 (1.9, 3.0)
SRS *t* score, per 10-point increase	1.2 (1.1, 1.3)	1.3 (1.2, 1.3)	1.4 (1.2, 1.5)	1.3 (1.2, 1.5)	1.4 (1.2, 1.7)	1.2 (1.1, 1.2)
Reported ID	Ns	ns	Ns	.6 (.4, .9)*	ns	ns
History of skill loss^a^	.8 (.6, 1.0)*	ns	.7 (.5, 1.0)*	ns	ns	.7 (.5, .8)

PDD-NOS= pervasive developmental disorder, not otherwise specified; AD/HD or ADD = attention-deficit/hyperactivity disorder or attention-deficit disorder; SRS = Social Responsiveness Scale (see Methods); ID = intellectual disability.

^a^Moderate to severe loss of communication and/or social skills between ages of 12 and 36 months.

^†^Adjusted for age; region (West versus all other); ethnicity; maternal education and history; evaluator.

*P < .05.

## Abbreviations

AS: Asperger syndrome;
ASD: Autism spectrum disorder;
SES: Socioeconomic status;
ID: Intellectual disability.
